# The Physiology, Pathology, and Treatment of Asphyxia; Including Suspended Animation in New-Born Children, and from Drowning, Hanging, Wounds of the Chest, Mechanical Obstructions of the Air-Passages, Respiration of Gases, Death from Cold, &c.

**Published:** 1834-10-01

**Authors:** 


*The Physiology, Pathology, and Treatment of Asphyxia; including suspended Animation in New-born Children, and from Drowning, Hanging, Wounds of the Chest, Mechanical Obstructions of the Air-Passages, Respiration of Gases, Death from Cold, &c. &c.* By James Phillips Kay, m.d., formerly President of the Royal Medical Society, Edinburgh.—London, 1834. 8vo. pp. 344.

How often has it been objected to the zealous cultivators of physiological knowledge, that they are in pursuit of a phantom, an ignis fatuus, which ever and anon they fancy they can seize on, but which incessantly eludes their grasp! The notion which we hear so often expressed, namely, that physiology must ever be an imperfect science, is no doubt well calculated to console the indolent, who will not take trouble to inquire even into the results of the labours of contemporaries. It were well if this doctrine had its influence only on such hybernating animals, and still better if it tended to increase the period of their torpidity; but it is unfortunate that even the opinions of such men have weight with a portion of the community, and it sometimes happens that they from whom we might expect better are entangled in their nets. Hence the outcry about the nonadvancement of physiological science is really often in the mouths of those who ought to speak differently. We do not mean to assert, that the advance of physiology has been commensurate with that of some other branches of natural science; but we do assert, (and we do so, fully aware of the opposition which the assertion will receive from many quarters,) that, taking into account the almost insurmountable obstacles which physiology has had to contend with, both as regards the nature of its objects, and also, we must add, the qualifications of the majority of its cultivators, this science has been by no means so tardy in its progress as many would have us to suppose. That which we most lament in connexion with physiology, is the great dearth of *well-qualified* labourers in her rich fields. We say well qualified; for no science possesses a more abundant supply of unskilful workmen,—mere dabblers, who, for want of something better to do, deem themselves privileged to retail the opinions of their predecessors, which they do often not in the purest and most unadulterated form. Of such physiologists we have enough; but of the zealous yet cautious men of science, who value facts above hypotheses, and know how to assign to both their respective positions,—of such men physiology stands much in need; nor can we expect it to make any very considerable advances from its present position, till a few such workmen are employed about it.

The foregoing reflections have resulted from the consideration that the subject of the work before us, asphyxia, is dependent for its elucidation entirely upon accurate physiology, and upon the precise knowledge of a function which it is not easy to investigate. In examining the function of respiration, there are numerous objects requiring very minute scrutiny before a single conclusion can be formed. Any one at all acquainted with the mass of matter that has been collected relative to this function, must be aware also of the great discrepancy that exists as to the statement of the results of experiments, and the differences in opinions which are consequent upon that: yet we have, in the work of the distinguished Edwards, "sur l'Influence des Agens Physiques, &c." an example of what the truly philosophic mind can effect, even amid such discordant elements: by his reasonings and experiments he has to a very great extent reconciled the conflicting conclusions of his predecessors, and established most of the important points connected with this function on a sure foundation.

Dr. Kay, in the work before us, commences by establishing the exact meaning and import of the term asphyxia, and its difference from syncope; both these states exhibiting phenomena very similar, but the former being, "by modern usage, employed to distinguish that form of pulselessness which is produced by any cause preventing the decarbonization of the blood, accomplished by respiration." According to him, asphyxia is to be regarded as a condition in which, "though the action of many organs has been changed, so as to be incapable of giving evidence of life by its natural phenomena, an action continues which supports a mode of life peculiar in its phenomena, and sometimes so long, that, by the use of means to restore the function of the lungs and circulating system, the natural actions may be revived." (P. 71.)

Our author offers an ingenious distinction between death and asphyxia, but, while we admire its ingenuity, we do not profess to subscribe to its truth.

"The death of the body, as distinguished from the organic death of the tissues, consists in the disunion of the intellectual principle from the corporeal organization.

"This connexion is maintained by various peculiar intestine motions in the various organs, but many facts indicate that the brain is the intimate residence of this mysterious tenant. The intestine actions of the brainular mass may be primarily or secondarily affected, that is, by the previous lesion of other organs, or without their lesion; but, until these organic motions become such as to disunite the immaterial intelligence, life continues, though none of the usual evidence of its existence be given by phenomena of which the senses can be cognizant. But, as we cannot conceive that, when once the union between mind and matter has ceased, the intellectual principle can be recalled to the corporeal organization, all states in which resuscitation is said to take place must be only apparent death, and the revival consists in a change from those morbid actions which suspended the usual phenomena of life, to those natural organic notions by which its usual functions are supported.

"Asphyxia differs from death, therefore, as in it the minute actions of all tissues continue such as to support the connexion with the intellectual principle. Although in death, as after decapitation, (when we cannot conceive the intellectual principle to be connected with the corporeal organization,) contractility and nervimotion long survive, and minute organic movements are continued, yet they have lost that portion of the organization which maintains the union with the intellectual principle. Whilst life yet remains, the minute actions may be so morbid that no means can revive those by which life can be supported for any period, and then death is certain. But the separation of the intellectual principle from the corporeal organization may take place long before all motion has ceased, and then death has ensued; yet, as life may be restored in the early stages of asphyxia, it follows that death has not happened, and that this state must be regarded as life peculiar in its phenomena." (P. 74.)

In speaking of the causes of asphyxia, our author notices that form of it which, in these days of locomotive enterprize, ought to be extensively known: we allude to that from rarefaction of the air at high elevations, and which is analogous to that produced artificially, by gradually exhausting a receiver in which an animal has been placed.

"Some travellers who have ascended very high mountains have suffered much from the rarity of the atmosphere near their summits. They have been unable to walk more than a few yards without stopping to take breath, and feeling extreme fatigue. This lessness has sometimes been accompanied by hemorrhage from the lungs, sudden exhaustion of the muscular power, and fainting.

"These alarming symptoms are produced by the want of a sufficient supply of oxygen to the lungs, whilst at the same time the pressure of the atmosphere on the capillary vessels is diminished, and a condition of the circulation produced, such as will be described when we treat of the physiological consequences of suspended respiration. Asphyxia from void has this peculiarity, that the capillary vessels of the whole surface of the body are congested from the removal of the atmospheric pressure on them, and particularly that, in the delicate and very vascular structure of the lungs, this congestion becomes so great as frequently to occasion hemorrhage, and always to interfere with the effect of the air on the blood circulating in that organ. We may dismiss this form of asphyxia by observing, that no remedy can relieve it but a proper supply of air. When the danger of its accession is imminent, the best method of preventing its occurrence is to avoid everything which can accelerate the circulation, impede the motion of the chest, or prevent a supply of oxygen to the lungs and skin. Perfect rest, a recumbent posture, and free exposure to the air, together with the application of stimulants to the olfactory and respiratory nerves, are the remedies from which the greatest benefit may be expected." (P. 76.)

We recommend to all our readers, but more especially to those engaged in obstetric practice, the perusal of the remarks on asphyxia in newborn children, which immediately follow the passage last quoted. We think our author shows most satisfactorily that asphyxia in adults, and the same state in newborn children, occur from causes very analogous; namely, from the absence of the influence of that organ which gives to the blood its nutritious and vivifying qualities,—the lung in the adult, the placenta with the fœtus. In children thus asphyxiated, insufflation is the great remedy. Dr. Kay recommends it to be done by the bellows of Leroy, through a properly covered canula introduced into the trachea. Caution must be observed not to inflate the lungs too much, although, as Dr. Kay observes, there is less danger of injury in the fœtal than in the adult lungs. We lately saw the Indian-rubber bag ingeniously adapted to this purpose by Mr. Weiss, which we conceive a valuable addition to the surgical accoutrements of the accoucheur.

The second chapter of this work contains a valuable abstract of Edwards' experiments and conclusions respecting the influence of air and water, and of venous blood, on the nervous and muscular systems; the relative power of animals of various ages to produce heat, and the causes of the loss of temperature with hybernating animals; on the influence of external temperature on young animals deprived of air; and the comparative necessity for a supply of oxygen which is experienced by different animals. The author then proceeds, in the third chapter, to inquire into the circumstances which cause a cessation of the contractility of the heart and muscles, when respiration is suspended in warm-blooded animals.

Every one knows how much diversity of opinion, and what angry controversy have originated in the attempt to explain the phenomena of death from asphyxia. From the days of the immortal discoverer of the circulation, this subject has justly attracted the attention of physiologists. We find a unity of sentiment between this great man and Haller on the subject; both of them attributing all the phenomena to a stoppage in the pulmonary circulation, consequent on the compression of the vessels of the lungs during expiration. This was an opinion suited to the days of the mechanical physiologists: its fallacy, however, was exposed by our countryman, Goodwyn, who shewed that the lungs did not collapse to such an extent as to compress their capillary vessels; but that the air remaining in them, even after the fullest expiration, distends them sufficiently to permit the blood to circulate freely through them.

Bichat's well-known experiment of adapting a stopcock to the trachea of an animal, so as to command the ingress of air, and then opening the carotid, fully proved the alteration which the blood undergoes in its passage through the lungs, and the necessity for inspiration, in order to affect that change: he remarked, moreover, not only a change in the physical characters of the blood, but also a great diminution in the force of its saltus from the divided artery, which was again increased on the readmission of the air. Dr. Kay repeated his experiment, with a view to ascertain how long the flow of blood continued during the privation of air; and, having varied it so as that the cessation of the flow of blood could not be attributed to the extent of the previous hemorrhage, he found that, although the heart continued to act vigorously, yet the flow of blood ceased in from three to five minutes after the animal was asphyxiated. It is evident that the cessation of the flow of blood was attributable not to any want of energy in the heart, but to the non-arrival of blood to the heart from the lungs; and this retardation in the pulmonary circulation was evidently owing to the want of inspiration, as might be deduced from the celebrated experiment of Hooke, which is similar to that of Bichat.

The question evidently, then, is reduced to this, what is the obstacle to the pulmonary circulation? Dr. Kay found that asphyxial blood could not be injected into the lung of an animal in asphyxia, with the same facility as arterial blood. The experiment by which he was led to this presumption (for it is not more,) was obviously one very difficult to perform; the injection of the lungs *in situ* in small animals being very difficult at all times, but more particularly so during life: yet the point is one not to be passed over, and which strongly favours the supposition that the vessels resisted the ingress of the noxious asphyxial blood, while they offered no obstacle to the arterial.

Goodwyn's theory of asphyxia rested on the hypothesis that the left auricle became distended with asphyxial blood, which was incapable of stimulating it to contraction; consequently, the blood coagulated in it, and the contents of the pulmonary veins could not find ingress into the auricle. Could this supposition be established, we need seek no further for an obstacle to the pulmonary circulation; but, unfortunately for it, it appears that the cessation of the heart's action is the last of the phenomena; that it takes place subsequently to the cessation of the flow of blood, and therefore cannot be its cause. This is proved not only by Dr. Kay's experiments, but by those of Bichat, who further shewed that dark blood might be injected into the left cavities of the heart, without destroying their contractility; and even that the contractility may be re-established, by injecting dark blood into the heart. It is clear, therefore, that Goodwyn's theory will not account for the stoppage of the pulmonary circulation.

Bichat admitted the continuance of the circulation of dark blood, for it was proved by his own experiments; but, because the voluntary muscles are greatly enfeebled, and almost paralysed, by the injection of asphyxial blood, he inferred that *all* tissues injected with such blood are similarly affected as to their vital properties, and therefore that the heart's functions were destroyed by the noxious qualities of this dark blood. This question has been well met by Dr. Kay, and his experiments satisfactorily shew that the non-contractility of the muscles is the result, not of the noxious influence of venous blood, but of their being deprived of their wonted supply. This view is still further confirmed by the fact, that if the main artery of a limb be tied so as to prevent the ingress, and the principal venous trunk be tied, so as to prevent the egress of the blood, the contractility of the muscles will continue longer than if the artery alone were tied. "The power of contraction," concludes our author, "is therefore in the direct ratio of the quantity of blood circulating in the muscular tissue, and its capacity is determined by the facility and velocity of that supply. When it is abstracted, contractility ceases. Blood is therefore necessary to contraction."

Further experiments very satisfactorily proved that a muscle, thus deprived of its contractility by the cutting off of its proper supply of blood, could be revived by the transfusion of blood; and that, although arterial blood was more favourable to the resuscitation, yet venous or asphyxial blood had also that effect to a certain extent; or, to state the conclusion in the author's own words, "dark blood is therefore less favourable than arterial to the contractility of muscles, but *its presence in the tissue* supports this power a considerable period after the ligature of the artery." This conclusion appears to us to be so completely established by well planned and judiciously varied experiments, that we cannot hesitate to give our fullest assent to it. How far, however, our author is warranted in stating that his experiments prove that "neither asphyxial or venous blood have *any noxious* influence upon the contractility of the voluntary muscles," we are not prepared to decide. We see no facts to warrant such a conclusion: *a priori*, we should say that any kind of nutritious fluid, different from the usual and healthy, would be more or less noxious; and we do not see that the fact which we admit as proven, viz. that a supply of venous blood supports muscular contractility, at all invalidates that supposition.

The right auricle of the heart has been long known as the *ultimum moriens;* it has also been observed, that the other cavities of the heart are not synchronous as to the cessation of their vital phenomena: to account for this, physiologists deemed it necessary to attribute to the heart peculiar properties, distinct from those of other muscular fibres. Nysten's observations on this subject are pretty generally known, and well deserving of attention; but Dr. Kay has succeeded in explaining the supposed peculiarity of the heart's contractility, by the application of the fact just established, viz. the relation of the supply of blood with the permanence of contractility. By experiment he could vary this permanence, so that the left auricle should become the *ultimum moriens*, if the right were deprived of its wonted supply of blood. Haller found that he could transfer the permanence of contractility from the right auricle to the left, by completely emptying the right side of the heart, leaving the pulmonary veins free, and tying the aorta, so that the left heart was gorged, while the right was completely empty. He ascribed the permanence of contractility to the presence of the blood, which he regarded as the stimulus in obedience to which the cavities contracted. Dr. Kay's experiments were similar to that of Haller, and he thus reasons upon them.

"The result of these experiments is perfectly consistent with the idea that the contractile power of the parts is supported, through a comparatively longer period, by the repletion of their cavities, and consequent congestion of their fibres with venous blood. This repletion of the right auricle, and of its venous sinus, in death, from the cessation of the functions of the brain or lungs, must be more considerable than that of any other muscle of the body. The blood, which by the last contraction of the ventricle is propelled into the arteries, is by them conveyed to the capillaries in the various tissues of the body. The organic motions of these tissues continue for some time; the blood still moves onwards, and is impelled with a certain force by their actions into the general venous system. The veins are everywhere found distended, because the circulation is obstructed by the lungs, in which respiration has ceased. The pressure of this power must be divided between the terminations of the large veins in venous radiculæ in all the system, and their opposite terminations in the heart. The power, therefore, will be greater in the auricle than in any other portion of the body; and, if congestion support the muscular power, that of the auricle ought to survive longer than that of any other organ, as experiment proves that it does." (P. 165.)

It is hardly necessary to remark how signal has been the overthrow of Bichat's hypothesis: it is clearly proved, by the experiments of our author, that the circulation of asphyxial blood, which, according to Bichat, paralysed the heart, had really an exactly opposite effect.

It appears, then, that the question, whence arises the obstruction to the pulmonary circulation in asphyxia? cannot be satisfactorily answered, either by the hypothesis of Goodwyn, or by that of Bichat; for, in fact, the pulmonary circulation ceases before the heart's action ceases. The obstruction cannot therefore be extraneous to the lung; it is not produced by any mechanical compression of the pulmonary vessels, or indeed by any mechanical cause whatever in the lungs, as both Goodwyn and Williams have established: but there seems no just ground for rejecting Dr. Kay's conclusion, "that the circulation is arrested after respiration ceases, because, from the exclusion of oxygen, and the consequent non-arterialization of the blood, the minute pulmonary vessels, which usually convey arterial blood, are then incapable of conveying venous blood, which therefore stagnates in the lungs."

It will be recollected that Bichat attributed the phenomena of asphyxia not merely to the circulation of dark blood in the substance of the heart, but to the absence of the usual venous influence occasioned by the circulation of the blood in the brain; but Dr. Kay has shewn that Bichat's experiments were so clumsily executed as to be wholly inconclusive, and, by very well devised experiments of his own, he has proved "that dark blood may circulate through the cerebral mass, without producing, by its contact with the brain, a sudden suspension of the functions of the nervous system." We have not space to follow the author through his experiments and reasonings on this subject, but we conceive that he has fully disproved Bichat's assertion, by shewing not only that the venous blood is not so noxious as that physiologist supposed, but also that, in asphyxia, it is transmitted in very small quantities to the brain: in fact, that the mischief is done before the dark blood can well be said to *circulate* in the cerebral mass.

We have an interesting chapter on the effects of the admission of air into the cavities of the pleural membranes. The novel, and we must say rather absurd, proposal of Carson, to procure rest to a lung by admitting air into the sac of the pleura, so as to compress it, was among the first circumstances to draw attention to this subject; and perhaps the most unreasonable part of Carson's proposition arises from his expectation that the quiescence of the lung would promote the absorption or removal of tubercular deposits. This fanciful notion, however, was soon set aside, when it appeared, from the very important experiments of Dr. Williams, of Liverpool, that it is not quite so easy to produce a collapse of the lung as it might be at first supposed; that, if the wound be moderate, the column of air entering the trachea is sufficient to counteract the pressure of the air entering the chest, and thus to prevent collapse; but if the wound be of considerable size, and so open that the air has very free ingress, then the compression is complete, and the animal so circumstanced will die.

This subject is one of much interest, as regards the mechanism of respiration. The phenomena of expiration are to a considerable extent explained by observing the effects of the admission of air into the thoracic cavity. By freely opening one side of the chest of an animal, so as to produce complete collapse of the lung, the action of the lung of the opposite side may be seen through the mediastinum, provided that the septum has been uninjured. This action is in no way impeded by separating the attachment of the abdominal muscles from the margin of the ribs, but the diaphragm continues to ascend, following the contraction or diminution of the lungs. That the contractile or elastic power of the pulmonary tissue is a principal agent in this, we can readily believe; but that the pleural membrane possesses a "resilient power," such as Dr. Kay ascribes to it, we think wants proof. A perforation of the mediastinum, so as to admit air into the cavity of the pleura, at once puts a stop to the action of the lung; it collapses, the diaphragm ceases to ascend: what further proof do we need that the lungs themselves are the primary agents in respiration?

Thus far we have endeavoured to give our readers a glimpse of the principal points of physiological, and therefore of pathological import, which Dr. Kay has embodied in his work. It will be observed that he differs from his predecessors in his theory of asphyxia, the phenomena of which he thinks are sufficiently accounted for by the stoppage of the pulmonary circulation. He seeks not for further aid from the nervous influence and other mysterious agents; and, as we are of opinion that he has fully established his views, we conceive him entitled to great merit in having so far simplified the theory of this condition.

The author devotes two chapters to the consideration of the various means which have been adopted for the purpose of recovering the asphyxiated. Of these means, insufflation is deservedly entitled to much consideration, as well from its real value as a resuscitating agent, as from its dangerous tendency when incautiously or injudiciously applied. Leroy's experiments shewed that air, introduced with even moderate force, produced bad consequences in rabbits, foxes, goats, sheep, and other animals: some were even killed by the inflation, and others suffered great dyspnœa for a considerable time afterwards. The most frequent result of insufflation is the escape of air from the ruptured pulmonary vesicles into the sac of the pleura, thus producing a condition favourable to asphyxia: in fact, Leroy and Magendie revived some animals thus treated, by making a puncture in the walls of the chest, and thus permitted the effused air to escape into the cavity of the pleura. Sometimes air was found throughout the sanguiferous system, and the several forms of pulmonary emphysema has been produced.

"Leroy recommends the double-valved bellows of Hunter, to the handles of which he has adapted the graduated arc of a circle, by which the quantity of air injected into the lungs, and removed by each successive motion of the bellows, may be determined."—"He ingeniously measured with his bellows the quantity of air expired without effort into a bladder at different ages, and marked upon the arc of the circle attached to the apparatus the point to which the elevated handle of the bellows was raised. The quantity of air to be introduced into the chest at successive ages being thus determined, he reduced the opening at the extremity of the curved tube to a size which prevented the air being introduced more rapidly than it is usually inspired. The diameter of the tubes, and of their orifices, were adapted to the ages of the patients, and the quantity of air which ought to traverse them in a given time was thus regulated." We readily agree with Dr. Kay in recommending this apparatus for general adoption, as, from its great simplicity, there can be no difficulty in using it; and it tends to diminish, if not to remove, the dangers attendant on the most important remedy we possess.

We cannot say so much for the simplicity of Leroy's method of applying galvanism, although it certainly is far preferable to that proposed by Dr. Ure, which requires an operation that can only be performed with safety by those who are possessed of a higher rate of anatomical knowledge than belongs to the ordinary race of medical practitioners in these days. Leroy introduced acupuncture needles into the diaphragm, (no easy thing in the human subject,) and connected the piles of a galvanic battery with them, so as to produce contraction of the diaphragm, and consequent enlargement of the abdominal cavity. By this method he succeeded on several occasions in reviving animals that had been under water more than five minutes. But a more simple method of imitating the natural respiratory motions is that effected by applying pressure to the abdomen and walls of the chest, alternated by a relaxation of that pressure. This is done by means of a many-tailed bandage, invented by Leroy; for a delineation and description of which we must refer the reader to Dr. Kay's work.

Dr. Kay strongly deprecates the having recourse to tracheotomy in asphyxia, and we think with great justice. It is unnecessary, as with common dexterity a curved canula can be introduced into the glottis; and Leroy has invented a useful instrument to facilitate this operation, by depressing the root of the tongue, and drawing the epiglottis upwards and forwards.

Much discrepancy of sentiment exists as to whether, in asphyxia from submersion, the water is inspired into the trachea. This subject is very fully discussed by our author: he seems to incline to the opinion that no water enters the trachea so long as the organic sensibility remains; but, if the body remains submerged after that has ceased, then it is natural to expect that it shall enter at every inlet: hence an argument may be drawn in favour of the extreme importance of an early removal of bodies from the water.

The question as to the efficacy of the transfusion of arterial blood into the veins in asphyxia, led our author to devote his seventh chapter to a very interesting and well-told history of the operation of transfusion. Its particular application to asphyxia is however still in its infancy, and requires further investigation.

The remainder of the work is occupied with the consideration of asphyxia from suspension, and death from cold, as well as the effects of the respiration of the noxious gases. Our limits prevent us from attempting to present the reader with any analysis of the valuable matter contained in the two last chapters. We must content ourselves with recommending most strongly to his perusal, not only these chapters, but also the whole work, as one containing a vast mass of information on the most interesting subjects, and also as an example of a calm and very philosophic discussion of some very difficult physiological points. Such works should be perused by medical men with a twofold object: first, as giving a practical lesson in the art of reasoning; and, secondly, from the increase which they may afford to the existing stock of knowledge.

The letter prefixed to this work, and addressed to the Duke of Northumberland, as president of the Humane Society, contains a useful abstract of all that is connected with the pathology and treatment of the asphyxiated. It ought to be printed in a cheap and separate form, and circulated far and wide throughout the country.

We take leave of Dr. Kay's work not without some regret. We would we could always ensure ourselves so agreeable a task in the business of reviewing as that we have had with this book. We trust this is not the only subject on which the talented author means to make his views and researches public: however that may be, we conceive he has done enough to entitle him to the warmest praise of his contemporaries, and to secure for himself a fame of no short duration.

